# A Machine Learning Approach for Detecting Digital Behavioral Patterns of Depression Using Nonintrusive Smartphone Data (Complementary Path to Patient Health Questionnaire-9 Assessment): Prospective Observational Study

**DOI:** 10.2196/37736

**Published:** 2022-05-16

**Authors:** Soumya Choudhary, Nikita Thomas, Janine Ellenberger, Girish Srinivasan, Roy Cohen

**Affiliations:** 1 Research Behavidence Inc New York, NY United States; 2 Data Science Behavidence Inc New York, NY United States

**Keywords:** mobile phone, depression, digital phenotyping, digital mental health

## Abstract

**Background:**

Depression is a major global cause of morbidity, an economic burden, and the greatest health challenge leading to chronic disability. Mobile monitoring of mental conditions has long been a sought-after metric to overcome the problems associated with the screening, diagnosis, and monitoring of depression and its heterogeneous presentation. The widespread availability of smartphones has made it possible to use their data to generate digital behavioral models that can be used for both clinical and remote screening and monitoring purposes. This study is novel as it adds to the field by conducting a trial using private and nonintrusive sensors that can help detect and monitor depression in a continuous, passive manner.

**Objective:**

This study demonstrates a novel mental behavioral profiling metric (the Mental Health Similarity Score), derived from analyzing passively monitored, private, and nonintrusive smartphone use data, to identify and track depressive behavior and its progression.

**Methods:**

Smartphone data sets and self-reported Patient Health Questionnaire-9 (PHQ-9) depression assessments were collected from 558 smartphone users on the Android operating system in an observational study over an average of 10.7 (SD 23.7) days. We quantified 37 digital behavioral markers from the passive smartphone data set and explored the relationship between the digital behavioral markers and depression using correlation coefficients and random forest models. We leveraged 4 supervised machine learning classification algorithms to predict depression and its severity using PHQ-9 scores as the ground truth. We also quantified an additional 3 digital markers from gyroscope sensors and explored their feasibility in improving the model’s accuracy in detecting depression.

**Results:**

The PHQ-9 2-class model (none vs severe) achieved the following metrics: precision of 85% to 89%, recall of 85% to 89%, F_1_ of 87%, and accuracy of 87%. The PHQ-9 3-class model (none vs mild vs severe) achieved the following metrics: precision of 74% to 86%, recall of 76% to 83%, F_1_ of 75% to 84%, and accuracy of 78%. A significant positive Pearson correlation was found between PHQ-9 questions 2, 6, and 9 within the severely depressed users and the mental behavioral profiling metric (*r*=0.73). The PHQ-9 question-specific model achieved the following metrics: precision of 76% to 80%, recall of 75% to 81%, F_1_ of 78% to 89%, and accuracy of 78%. When a gyroscope sensor was added as a feature, the Pearson correlation among questions 2, 6, and 9 decreased from 0.73 to 0.46. The PHQ-9 2-class model+gyro features achieved the following metrics: precision of 74% to 78%, recall of 67% to 83%, F_1_ of 72% to 78%, and accuracy of 76%.

**Conclusions:**

Our results demonstrate that the Mental Health Similarity Score can be used to identify and track depressive behavior and its progression with high accuracy.

## Introduction

### Background

The American Psychiatric Association defines depression as a “common and serious medical illness that negatively affects how you feel, the way you think, and how you act” [1]*.* It comprises symptoms such as low mood, guilt, suicidal ideation, and cognitive decline [1,2]. According to *The Global Burden of Diseases, Injuries, and Risk Factors Study (GBD)* 2019, depression is one of the most disabling mental health disorders [3], and it poses a significant economic and medical burden. A study by Greenberg et al [4] calculated an increase in economic cost related to depression of 37.9% from US $236.6 billion to US $326.2 billion in 2020. These costs comprised direct, suicide-related, and workplace costs [4]. There also has been a global increase in the prevalence of depression. The percentage of adults in the United States with major depressive disorder increased by 12.9%, from 15.5 to 17.5 million, between 2010 and 2018 [4]. To further add to these increasing numbers worldwide, the COVID-19 pandemic has led to a substantial increase in mental health conditions, including depression [5], which has been aggravated by the uncertainty associated with the disease, isolation, and overall decreased social interaction [6,7]. Given this rise in depression rates and the immense costs associated with it, adequate diagnosis and timely intervention have become a pressing and urgent need [8].

Depression, as most other mental illnesses, is diagnosed via the Diagnostic and Statistical Manual of Mental Disorders, Fifth Edition (DSM-5) [9], or the International Classification of Diseases, 11th Revision [10]. However, there is growing skepticism regarding their validity [11,12]. In a groundbreaking research study by Newson et al [11] in 2021, they were able to quantify the degree of heterogeneity within and across the DSM-5 symptom profile in that the DSM-5 criteria “fails to diagnose *patients* by symptom profile any better than random assignment.” This strongly supports Zimmerman et al [13], who found that there are 227 different ways to diagnose depression. The problem is further exacerbated by heterogeneity among scales used for depression screening and diagnosis [14,15], illustrated by a cross-sectional study that found that, in a small sample of 309 patients, there was a misdiagnosis in 55% of these cases [16]. In addition, there are no approved biomarkers as part of the diagnostic criteria for depression [17]. Compounding factors that contribute to the hurdles associated with adequate screening and monitoring of depression are lack of primary care physicians, low recognition of depression in primary care [18], delayed response to treatment [19], 12-week waiting period in the absence of a response, other comorbidities, and patient fear of stigma attached to depression [20]. There are several instruments for detecting depression in primary care [21], one of which is the Patient Health Questionnaire-9 (PHQ-9), which has been adopted as the gold standard for detecting depression and grading its severity [22].

To overcome these many challenges associated with traditional methods for the detection, management, and monitoring of depression, smartphone-based interventions have advanced as an available and alternate option. Middleweerd et al [23] found that the use of digital tools for physical health monitoring, such as fitness-based smartphone apps, was becoming increasingly popular. The use of digital tools for the management of mental health conditions became a key resource as the demand for mental health support exceeded the supply when the COVID-19 pandemic led to a rise in depressive disorders worldwide [6]. In addition to telehealth and remote therapy, a solution that emerged was digital health assessment using smartphones and their sensors [24], also known as digital phenotyping. Torous et al [25] define the term as “moment-by-moment quantification of the individual-level human phenotype in-situ using *data* from smartphones and other personal digital devices*.*” The use of passive sensors in the mental health industry has the potential to detect real-time changes in psychological factors, and this can be used to increase access to care [26], reduce stigma [27] improve diagnosis [28], and enable remote monitoring [29] as has been established by previous and ongoing research.

### Previous Work

There is a growing body of research on passive data sensing and its use in modeling human behavior [30]. Previous work has shown that monitoring these digital biomarkers using machine learning models to assess passive smartphone data can aid in the screening, treatment, and remote monitoring of mental health disorders. In a study by Wang et al [31], the app Student Life was used to show the correlation between depression and accelerometer- and screen use–based biomarkers. In another study, Saeb et al [32] found significant correlations between depression and passive data such as phone use and GPS in a sample of 40 participants. Asare et al [33] found that age group and gender as predictors led to improved machine learning performance. Their study concluded that behavioral markers indicative of depression can be unobtrusively identified using smartphone sensor data [33]. Taking a machine learning approach, a study found that the predictive power of mobile device use patterns was significant to continuously screen for depressive symptoms or monitor ongoing treatments [34]. In line with this study, another study used the Remote Monitoring Application in Psychiatry to explore the validity of smartphone-based assessments for self-reporting mood symptoms and found high compatibility with nonsmartphone-based assessments [35], thus proving such tools to be helpful for clinicians and research.

This study focused on South Korea as it has consistently reported a low number of depression cases despite high suicide rates [36] and the dramatic worldwide increase in depression. In 2005, a study reported that the annual prevalence rate of depression in South Korea was 1.7%, whereas rates of depression were reported to be much higher in that 25.3% scored positive for depression in a nationwide sample study [37]. Researchers have debated various reasons for the low prevalence rates, including the difference in cutoff scores in South Korea versus other countries and the associated stigma [37] attached to mental health conditions, but have not yet reached a conclusion as to the cause. Digital phenotyping and passive monitoring can provide a timely opportunity to target issues such as low access to mental health diagnoses, stigma, and associated health consequences in South Korea and similar countries.

### Objective

Smartphone sensors and passive data, when coupled with relevant statistical and machine learning models, provide an avenue to capture behavioral changes associated with mental health disorders in naturalistic settings [30,38]. Much of the previous work in this field has used sensors that are invasive and privacy-related such as GPS, call logs, SMS text message logs, and keyboards. This study demonstrates a novel mental behavioral profiling metric termed Mental Health Similarity Score (MHSS), derived from analyzing passively monitored nonintrusive and nonidentifiable smartphone use data, to identify and track depressive behavior.

## Methods

### The Study Design

**Figure 1:**
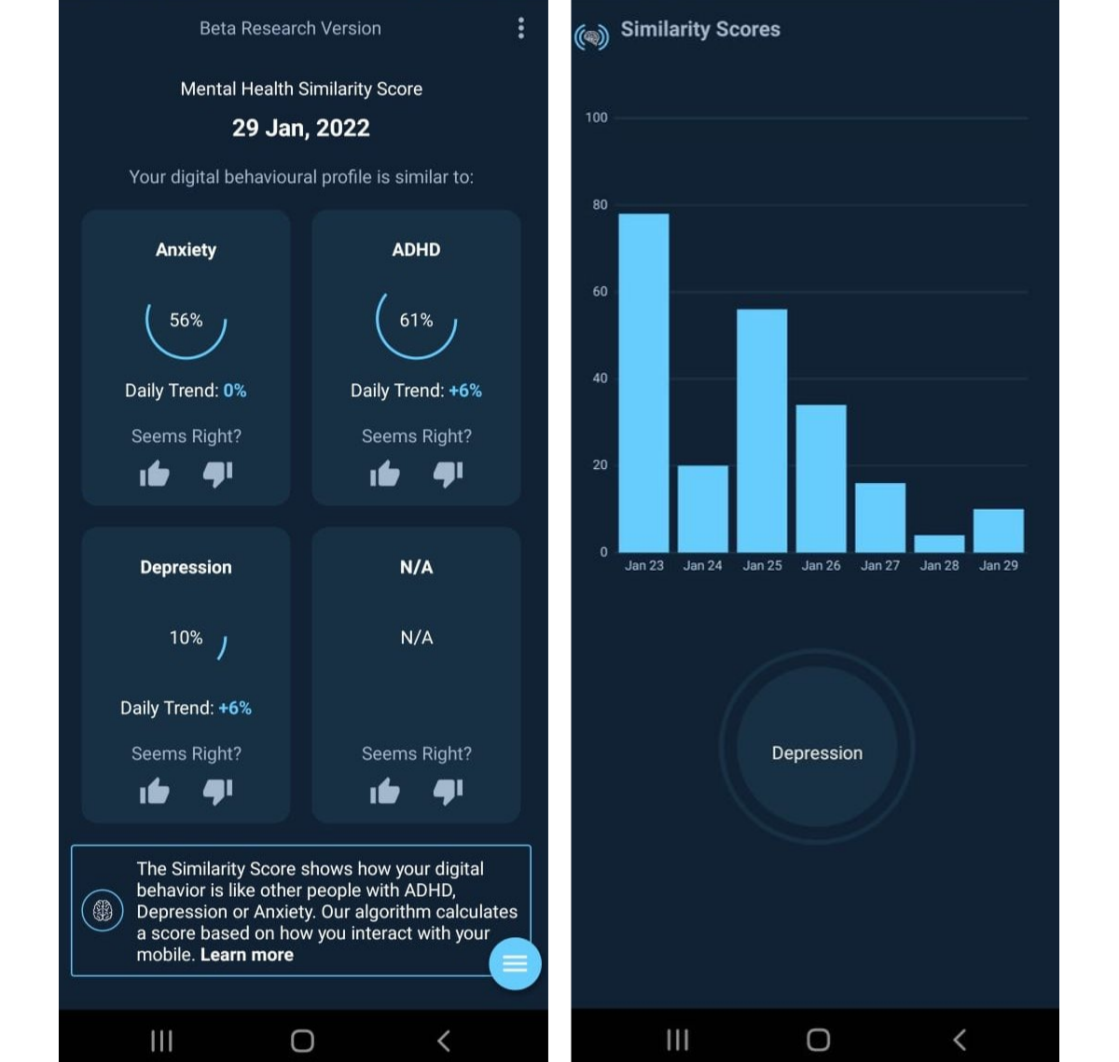
The Behavidence app screen showing the daily Mental Health Similarity Score.

We collected active and passive data in a longitudinal observational study using the Behavidence (Behavidence, Inc) mobile app, derived from a cohort of anonymous participants in South Korea. Participants were invited to take part in this study through social media advertisements and campaigns, which is an effective tool for recruitment in research studies [39]. The advertisement used a research code that the interested individual could use to enter the study by downloading the app (Figure 1). The data set was collected from 558 participants between November 2021 and December 2021. All the participants were Android-based smartphone users.

### Participant Inclusion Criteria

The inclusion criteria were as follows: (1) they must be aged >18 years and (2) they must have an Android device.

### Measure (PHQ-9)

Depression severity was assessed using a patient-reported outcome measurement questionnaire. When the participants registered with a research code and enrolled in the study, they answered the PHQ-9 [40] in the app. The PHQ-9 scale is the gold standard for detecting and measuring the severity of depression worldwide [41]. It has been validated for use in community-based and general population settings and has sound psychometric properties [42]. Although the depression assessments were self-reported, the PHQ-9 has been clinically validated for the assessment of depression severity owing to its high internal reliability (Cronbach *α*=.89) and has been used in multiple studies as a self-reported questionnaire [22]. The PHQ-9 measures depression severity over the preceding 2 weeks. Each item of the PHQ-9 is scored on a scale of 0 (not at all) to 3 (nearly every day). The total PHQ-9 score ranges from 0 to 27, with a score of ≥10 indicating a major depressive disorder [22]. A score of <5 indicates no depression, 5 to 9 indicates mild depression, 10 to 14 indicates moderate depression, 15 to 19 indicates moderately severe depression, and 20 to 27 indicates severe depression [40]. The PHQ-9 has also been established to have good psychometric properties in the South Korean population, which is the focus of this study [43].

### Smartphone App (Behavidence)

Behavidence [44] is a mental health screening app that passively collects personal device use data with zero respondent burden and no use of identifiable information. The app works as an always-on solution and can be downloaded from the Google Play store. Individuals can register or log in to the app with no supervision, and any required onboarding information was easily made available to the study participants remotely. In addition, demographic user profile information questions (gender, age bracket, and existing mental health indication) were answered within the app.

The Behavidence Research App was developed for smartphones running Android version 5 or higher and requires connectivity to send data to the back end for analysis and receive data analysis outcomes. It does not require connectivity to collect the data. For an app to run as a background process, it must obtain the *Battery Optimization* and *Usage Data Access* permissions from the user. These permissions are obtained during the onboarding process.

The app uses the principle of digital phenotyping to track user behavior. It displays an MHSS developed from phone use metrics such as time spent on various apps on a daily and weekly basis. The MHSS displays how similar the user’s digital behavior is to the digital behavior of someone who has been diagnosed with depression. This similarity score is a range from 0% to 100% (Figure 1). The MHSS is generated every 24 hours. The app also shows the user their weekly history of similarity scores. In this study, gyroscope readings from each participant’s smartphone device were collected in addition as sensor features measuring the direction and speed at which the phone was spinning around its axis.

The app and back end use strict data privacy and security protocols. The solution is compliant with the Health Insurance Portability and Accountability Act and the General Data Protection Regulation.

### Data Inclusion and Exclusion

The data set used for model training only included participants aged ≥18 years with at least 24 hours of complete passive, nonsensor, personal device use data. The final data set contained 399 participants with an average of 10 (SD 25.21) days of mobile data. The addition of the use of gyroscope readings, measuring angular velocity as phone sensor features, was tested to improve the accuracy of the model. The purpose of this is to test whether sensor features provide additional insights than using only nonsensor features. The data set used specifically for the gyroscope model training had a reduced individual participant number of 193, in which at least 24 hours of raw device use with the additional sensor readings was available. This reduced number of participants was due to the inability to collect gyroscope readings from specific Android phones.

### Feature Extraction

The raw data set collected contains daily behavioral patterns that were data-cleaned and transformed to reach independent features such as opening and closing apps with the start and end times in Coordinated Universal Time in milliseconds. These data were preprocessed by converting the time stamps to local dates and times according to the user’s time zone. Digital biomarkers, used as machine learning features in this study, were calculated per user, taking daily behavioral patterns on a 24-hour basis starting from midnight every day. The 3 main types of nonsensor features were *average time on the phone per day*, *frequency of events per day*, and *app category use per day.* The mapping of various apps into specific categories can be found in Multimedia Appendix 1. In addition, gyroscope data were collected and processed daily to generate sensor features such as *mean activity*, *average gap activity*, and *total activity.* In the end, a total of 37 features were extracted and merged on a per-user, per-day basis. Explanations of each feature can be found in Multimedia Appendix 2.

### Procedure

The participants downloaded the Behavidence app and answered a simple demographic questionnaire along with the informed consent form. They then completed the PHQ-9. The questionnaire was answered only as a 1-time data point and, thereafter, they were free to use the app on their own. The app generated a daily MHSS. The app added no further respondent burden and, therefore, the participants were able to check the score whenever they felt the need to or not at all.

### Data Analysis

#### Imbalanced Data Handling

A total of 24 hours of raw data each day were binned for every participant and considered separate observations in this study. Therefore, an individual with depression who had 10 days of complete 24 hours of passive data was considered as 10 depression-labeled observations. To correct for the imbalanced training data of the *none* and *severe depression* categories, the cohort with the smaller number of observations was randomly sampled to match the number of observations in the other. In this case, more observations were found in the *none* (ie, not depressed) group and, thus, it was randomly split into equal subsets. In addition, bootstrapping with 15-fold cross-validation was performed to assess the overall model performance.

#### Machine Learning to Predict Depression

A mental health profiling metric termed MHSS was derived from the features extracted from the raw data to classify whether a user’s daily digital behavior mimicked the digital behavior of mobile users who are depressed. This metric is a direct output of a machine learning model trained to classify 24 hours of digital behavior into the different thresholds of the PHQ-9, screening positive for severe depression versus no depression. A variety of machine learning models were compared to detect major digital behavioral differences between *none* and *severe* category participants. The algorithms tested in this study include random forest regression, multivariate adaptive regression splines, random forest classification, extreme gradient boosting, and support vector machines with a radial basis function kernel. After the top algorithm was chosen based on the highest predictive accuracy, 4 machine learning models were created and compared: the PHQ-9 binary nonsensor model, the PHQ-9 binary gyroscope sensor model, the PHQ-9 3-class model, and the PHQ-9 question-specific models.

The PHQ-9 binary nonsensor model was intended to classify participants who scored as severe (scores ≥20) on the PHQ-9 against those who scored as having no indication of depression (scores <5). The 3 main feature categories (average time on the phone per day, frequency of events per day, and app category use per day) were the main components input into this model. The PHQ-9 binary gyroscope sensor model had the same specifications as the PHQ-9 binary nonsensor model; however, 3 features (mean activity, average gap activity, and total activity) were added to the training to assess whether the gyroscope sensors had higher accuracy than the PHQ-9 binary nonsensor model. The PHQ-9 3-class model was intended to classify participants who scored as severe (>20), moderate (10-14), and no depression (<5) to help with predicting the progression toward severe depression. Finally, a model was built using specific PHQ-9 items that had the highest correlations with the nonsensor passive digital biomarkers to detect specific symptoms of depression rather than classifying them into *none* and *severe* categories.

#### Model Validation

The main metric used to validate the models built in this study tested whether most days of data collected had either high or low MHSSs. The training cohort was taken at a specific time point during the study’s recruitment in December and, thereafter, all additional days of data collected were used for the machine learning validation set. The metrics were tested on both a 1-week data majority and an overall majority on all days of data that were collected from the user by the app. If most days had high MHSSs, defined as having scores >50%, the user was classified as having depression. If most days had low MHSSs, defined as having scores <50%, then the user was classified as not showing signs of depression. This, in addition to the model accuracy and recall rates, will be used to assess whether digital biomarkers can detect and track depression.

#### Correlation Analysis

Further analysis of all items (questions) from the PHQ-9 was conducted to determine which symptoms of depression could be identified from the passive digital data collected through the app. A Pearson correlation and Spearman correlation were assessed to determine whether there was either a linear correlation or a monotonic relationship where the rate was not constant. Correlations were conducted on all 9 questions of the PHQ-9 scale with an MHSS as well as a combination of different questions.

#### Software

The Amazon Web Services platform was used for data storage, whereas data processing, feature engineering, model training, and poststatistical analysis were written in Python 3.8 programming language (Python Software Foundation). The packages used include pandas, stats models, and scikit-learn random forest classifier.

### Ethics Approval

Consent was voluntarily given on the participants’ smartphones once they were informed of the purpose of the study. The data set does not contain personally identifiable or any personal health information. The advertisement, informed consent, and study protocol were approved by the independent Western Institutional Review Board-Copernicus Group, Institutional Review Board (approval number: 20216225).

## Results

### Participants

Self-reported demographic data from the 558 participants (Table 1) show that, of these, 286 (51.3%) identified as women, 254 (45.5%) identified as men, and 18 (3.2%) identified as nonbinary or preferred not to disclose their gender. Regarding the participants’ age distribution, of the 558 participants, 474 (84.9%) were aged between 18 and 25 years, 29 (5.2%) were aged between 26 and 35 years, 42 (7.5%) were aged between 36 and 55 years, 10 (1.8%) were aged between 56 and 64 years, and 3 (0.5%) were aged ≥65 years. The PHQ-9 questionnaire was administered to users in both English and Korean, with most of the participants belonging to the Korean-speaking population (487/558, 87.3%).

**Table 1:** Demographic distribution showing the numbers for age, gender, and language of the answered Patient Health Questionnaire-9 (N=558).

Variable			Value, n (%)
**Age (years)**			
	18-25	474 (84.9)	
	26-35	29 (5.2)	
	36-55	42 (7.5)	
	56-64	10 (1.8)	
	>64	3 (0.5)	
**Gender**			
	Male	254 (45.5)	
	Female	286 (51.3)	
	Prefer not to say	18 (3.2)	
**Language**			
	Korean	487 (87.3)	
	English	71 (12.7)	

### Smartphone Data and PHQ-9 Distribution

Table 2 presents the distribution of the PHQ-9 scores of the 558 participants. The PHQ-9 was collected at the start of recruitment at a single time point during this study. The distribution of the PHQ-9 scores was as follows: 11.3% (63/558) were in the *none* category (ie, they were not depressed) with PHQ-9 scores <5, whereas 88.7% (495/558) showed signs of depression by scoring between *mild* and *severe*. The mean PHQ-9 score was 12.5 (SD 6.29).

There is an imbalance in the gender distribution when looking into each severity group of depression. For the *none* and *mild* cohorts, men represented the majority, whereas, in the *moderate*, *moderately severe*, and *severe* cohorts, there was a female majority, as shown in Table 3.

Out of the 558 participants, 499 (89%) answered “no previous diagnosis” in the demographic questions collected at onboarding. Moreover, 65% (323/499) of the participants who reported that they had no previous diagnosis of any kind obtained a PHQ-9 score of at least moderate (≥10) to severe depression, as shown in Table 4.

**Table 2:** Distribution of the participants’ PHQ-9^a^ scores (N=558).

PHQ-9 score category and score			Participants, n (%)
**None**			**63 (11.3)**
	0	20 (3.6)	
	1	6 (1.1)	
	2	12 (2.2)	
	3	7 (1.3)	
	4	18 (3.2)	
**Mild**			**124 (22.2)**
	5	13 (2.3)	
	6	33 (5.9)	
	7	23 (4.1)	
	8	37 (6.6)	
	9	18 (3.2)	
**Moderate**			**162 (29)**
	10	23 (4.1)	
	11	28 (5)	
	12	29 (5.2)	
	13	43 (7.7)	
	14	39 (7)	
**Moderately severe**			**134 (24)**
	15	29 (5.2)	
	16	31 (5.6)	
	17	26 (4.7)	
	18	31 (5.6)	
	19	17 (3)	
**Severe**			**75 (13.4)**
	20	16 (2.9)	
	21	16 (2.9)	
	22	13 (2.3)	
	23	6 (1.1)	
	24	10 (1.8)	
	25	5 (0.9)	
	26	1 (0.2)	
	27	8 (1.4)	

^a^PHQ-9: Patient Health Questionnaire-9.

**Table 3:** Distribution of gender among the PHQ-9^a^ scoring categories (N=558).

PHQ-9 category	Male, n (%)	Female, n (%)	Other or prefer not to answer, n (%)
None	41 (65.6)	21 (32.8)	1 (1.6)
Mild	69 (56)	52 (41.6)	3 (2.4)
Moderate	73 (45.1)	82 (50.6)	7 (4.3)
Moderately severe	48 (35.8)	82 (61.3)	4 (2.9)
Severe	24 (32.1)	47 (62.8)	4 (5.1)

^a^PHQ-9: Patient Health Questionnaire-9.

**Table 4:** Distribution of individuals who self-reported “no previous diagnosis” among the PHQ-9^a^ scoring categories (N=499).

PHQ-9 category	Participants, n (%)
None	63 (12.6)
Mild	113 (22.6)
Moderate	145 (29.2)
Moderately severe	119 (23.8)
Severe	59 (11.8)

^a^PHQ-9: Patient Health Questionnaire-9.

### Features Engineered From Smartphone Data

A total of 37 features were computed from the raw passive smartphone data. Of the 37 features, 29 (78%) showed statistical significance in the 1-tailed *t* test results between the *none* and *severe* cohorts. Overall, 8 of the significant nonsensor and gyroscope (sensor) features are displayed in Table 5. The remaining list can be found in Multimedia Appendix 3. Effect size analysis showed that the most important features had moderate to high effect sizes when comparing the *none* and *severe* category populations [45].

**Table 5:** The *t* test (1-tailed) results of the none versus severe cohorts with *P* values and Cohen *d* statistic.

Feature			Cohort, mean (SD)					*P* value			Cohen *d*
			None		Severe						
**Nonsensor**											
	Mean session time	1.1 (0.5)		2.5 (4.8)		<.001			0.4257		
	Total session	300.0 (145.0)		416.7 (233.3)		<.001			0.5811		
	Number of opens	305.7 (137.9)		240.8 (169.6)		<.001			0.4248		
	Sleep	266.7 (183.3)		300.0 (216.7)		<.001			0.1947		
	Average gap	3.2 (3.5)		4.3 (6.2)		<.001			0.2495		
**Gyroscope (sensor)**											
	Average activity	28.5 (67.5)		57.0 (101.0)		<.001			0.2053		
	Average gap activity	7.6 (13.9)		23.8 (47.5)		<.001			0.3191		
	Total activity	1181.6 (436.7)		1165.0 (446.7)		.68			0.0859		

### Predicting Depression From Features

Among the classification algorithms, random forest proved to have the highest predictive accuracy (87%). Extreme gradient boosting followed with an accuracy of 86%, whereas the support vector machine classifier had the lowest accuracy (44%), as shown in Table 6.

The top-performing algorithm, the random forest classifier trained on the PHQ-9 binary nonsensor model (none vs severe on the depression rating scale) on 34 of the nonsensor features mentioned in Table 5, achieved a precision of 85% to 89%, recall of 85% to 89%, F_1_ of 87%, and overall accuracy of 87%, as shown in Table 7.

**Figure 2:**
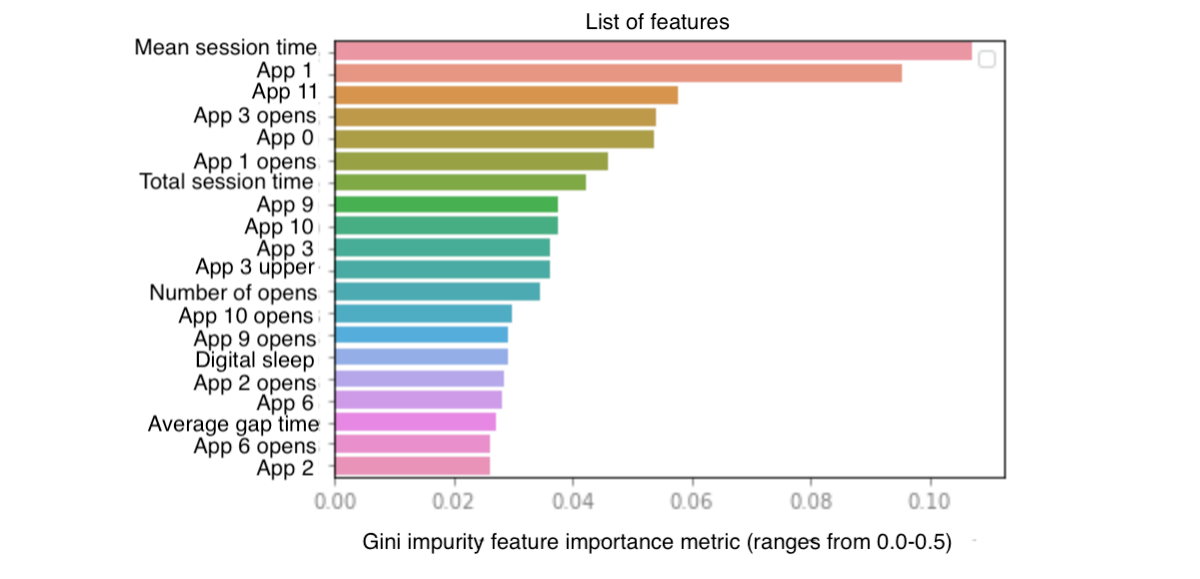
Feature importance plot of the Patient Health Questionnaire-9 binary nonsensor model achieving 87% accuracy. The x-axis represents the feature importance metric, Gini Impurity, which can range from 0.0 to 0.5. The y-axis represents the list of features ordered from greatest to least importance.

The feature importance plot based on Gini Impurity measurement analysis indicates the top passive digital features indicative of differentiating between *none* and *severe* cohort participants (Figure 2). The top 5 features are mean session time on the phone within a 24-hour period, average session time in the social interaction apps (app category 1), average session time within a 24-hour period in the miscellaneous and additional passive recreational apps (app category 11), number of times active messaging and communication apps (app category 3) were opened within a 24-hour period, and average time spent on unofficial or unregulated apps (app category 0). The top 5 features had Gini Impurity values ranging from 0.6 to 0.1.

The top 5 features from the feature importance list achieved statistical significance with *P*<.001, as shown in Table 8. Average overall session time and average time spent on social interaction apps, miscellaneous and additional passive recreational apps, and unregulated apps had greater mean values for the participants who scored as *severe* compared with the participants who scored as *none* on the PHQ-9. *None* participants opened active messaging and communication apps 110 times on average (SD 7.02), whereas *severe* participants opened this app category 74 times on average (71.05).

In addition, the model was tested to see whether it could accurately predict participants who had reported a previous diagnosis of depression. The model achieved an accuracy of 80% in detecting depression but only 26% in detecting the *none* group.

Additional validation of whether the PHQ-9 binary nonsensor-based model (none vs severe) could accurately predict the progression of depression was performed by calculating the percentage of participants in each group who had a majority of days with high MHSSs (>50%), with the MHSS as the model’s prediction of class probabilities. As shown in Table 9, the majority increases as severity increases, indicating that participants with severe depression had a majority of days with high MHSSs, supporting the model’s prediction ability.

When the gyroscope features were added as additional markers, the overall accuracy dropped to 76% with a precision of 74% to 78%, recall of 67% to 83%, and F_1_ of 72% to 78%. This model was also tested on the self-proclaimed diagnosis cohort and achieved 27% accuracy in detecting depression and 0% accuracy in detecting the *none* group. When age and gender were added to see if demographics played a role in classifying *none* (not depressed) versus *severe* (depressed) cohort participants, the overall accuracy decreased slightly from 87% to 84%, precision increased from 82% to 87%, recall increased from 83% to 86%, and F_1_ increased from 84% to 85%.

An additional random forest classifier trained on the PHQ-9 3-class model—none (PHQ-9 <5), moderate (10≤PHQ-9<15), and severe (PHQ-9≥20) depression on the 34 nonsensor features—achieved a precision of 74% to 86%, recall of 76% to 83%, F_1_ of 75% to 84%, and overall accuracy of 78%, as shown in Table 7.

**Table 6:** Accuracy metrics of the 3 classification algorithms tested in this study: random forest, extreme gradient boosting (XGBoost), and a support vector machine with radial basis function kernel.

Metric and cohort		Random forest model (%)	XGBoost model (%)	SVM^a^ model (%)
Accuracy		87	86	44
**Precision**				
	None	89	82	44
	Depression	85	90	0
**Recall**				
	None	85	90	100
	Depression	89	81	0
**F_1_**				
	None	87	86	61
	Depression	87	85	0

^a^SVM: support vector machine.

**Table 7:** Accuracy metrics of all models trained in this study.

Metric and cohort		PHQ-9^a^ binary nonsensor model (%)	PHQ-9 binary gyroscope (sensor) model (%)	PHQ-9 three-class model (%)	PHQ-9 questions model (%)
Accuracy		87	76	78	78
**Precision**					
	None	89	78	75	80
	Moderate	N/A^b^	N/A	86	N/A
	Severe	85	74	74	76
**Recall**					
	None	85	67	76	75
	Moderate	N/A	N/A	83	N/A
	Severe	89	83	76	81
**F_1_**					
	None	87	72	75	78
	Moderate	N/A	N/A	84	N/A
	Severe	87	78	75	89

^a^PHQ-9: Patient Health Questionnaire-9.

^b^N/A: not applicable.

**Table 8:** Mean values of each cohort and *P* values from 1-tailed *t* tests of the top 5 important features in the Patient Health Questionnaire-9 binary nonsensor model.

Feature	Cohort, mean (SD)		*P* value
	None	Severe	
Mean session time: average session length that a user interacts with their mobile device within a 24-hour period (minutes)	1.07 (3.58)	2.49 (4.97)	<.001
App 1: average time a user spent on apps that fall into app category 1—social interaction apps—within a 24-hour period (minutes)	1.41 (4.40)	3.58 (1.16)	<.001
App 11: average time a user spent on apps that fall into app category 11—miscellaneous and additional passive recreational apps—within a 24-hour period (minutes)	1.56 (1.94)	3.37 (5.11)	<.001
App 3 opens: number of times a user opened apps that fall into app category 3—active messaging and communication apps—within a 24-hour period (counts)	110.49 (70.10)	74.45 (71.05)	<.001
App 0: average time a user spent on apps that fall into app category 0—nonofficial or unregulated apps—within a 24-hour period (minutes)	0.34 (0.60)	0.83 (2.34)	<.001

**Table 9:** The PHQ-9^a^ binary nonsensor-based model validation results showing the majority of days with high MHSS^b^ across all days of data collected.

PHQ-9 severity	Participants, n (%)	Majority of days of data with MHSSs >50% (%)^c^
None	38 (18)	15.84
Moderate	116 (55.5)	75.02
Severe	55 (26)	95.82

^a^PHQ-9: Patient Health Questionnaire-9.

^b^MHSS: Mental Health Similarity Score.

^c^Each participant has a different total number of days of data collected. Hence, each PHQ-9 group has a different total number of days. Therefore, the majority of days mentioned is the total percentage of days that group participants had MHSS greater that 50%.

### PHQ-9 Specific Questions and Smartphone Data

A significant positive Pearson correlation was found among PHQ-9 questions 2, 6, and 9 within the *severe* category users and the mental health behavioral profiling metric (*r*=0.73), as shown in Table 10. When a gyroscope sensor was added as a feature, the Pearson correlation among questions 2, 6, and 9 dropped from 0.73 to 0.46.

**Figure 3:**
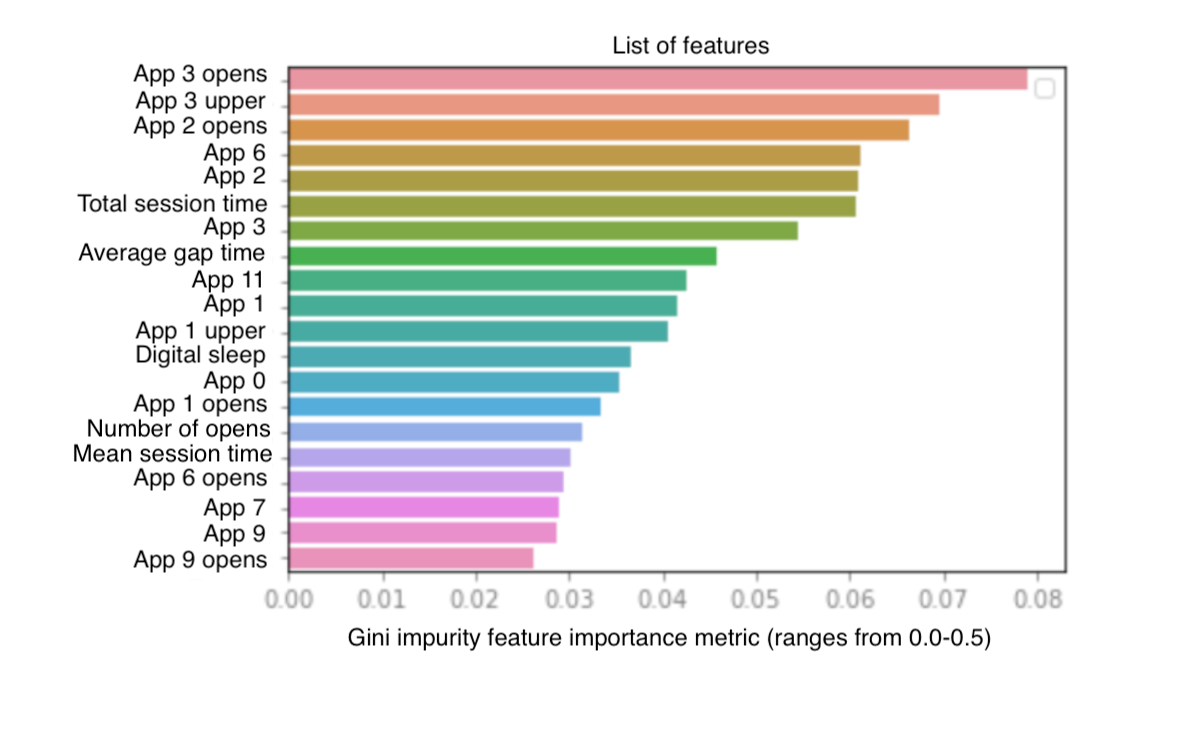
Feature importance plot of the random forest model for Patient Health Questionnaire-9 questions 2, 6, and 9. The x-axis represents the feature importance metric, Gini Impurity, which can range from 0.0 to 0.5. The y-axis represents the list of features ordered from greatest to least importance.

A binary model trained on questions 2, 6, and 9 was constructed to complement the PHQ-9 binary nonsensor model. The participants who scored 0 on all 3 questions were considered as the *none* class, whereas the participants who scored 3 on every question were considered as the *depression symptoms* class. The PHQ-9 questions model achieved an overall accuracy of 78% with a precision of 76% to 80%, recall of 75% to 81%, and F_1_ score of 78% to 79%, as shown in Table 7. Figure 3 shows the feature importance plots for this prediction model. Top features include (1) number of times active messaging and communication apps (app category 3) were opened within the 24-hour period, (2) number of times active messaging and communication apps were opened or longer than 1 SD from the mean session time within the 24-hour period (app 3 upper), (3) number of passive information consumption apps (app category 2) opened within the 24-hour period, (4) average time spent on general utilities apps (app category 6), and (5) average time spent on passive information consumption apps (app category 2).

Table 11 displays the mean values of the top 5 features of the random forest model for PHQ-9 questions 2, 6, and 9. The number of times the participants opened active messaging and communication apps that had greater session lengths than the average was calculated for both the *none* and *severe* participants and proved to be both statistically significant (*P*<.001) and a top feature in the questions model. The *none* participants opened this app category 6.47 times on average compared with the *severe* participants, who opened it 3.25 times. In addition, *none* participants opened passive information consumption apps 2.13 times on average compared with *severe* participants, who opened them 0.46 times on average. Finally, *severe* participants had general utilities apps opened for longer (0.57 minutes) on average than the *none* participants (0.40 minutes), but *none* participants had passive information and consumption apps opened for longer (0.29 minutes) on average than *severe* participants (0.18 minutes).

**Table 10:** Correlation analysis within the severe cohort between baseline per-item scores and Mental Health Similarity Scores on the day of baseline assessment.

PHQ-9^a^ item	Pearson correlation	Spearman correlation
Question 1: “Little interest or pleasure in doing things?”	−0.078	−0.075
Question 2: “Feeling down, depressed, or hopeless?”	0.596	0.607
Question 3: “Trouble falling or staying asleep, or sleeping too much?”	0.004	0.0
Question 4: “Feeling tired or having little energy?”	−0.101	−0.045
Question 5: “Poor appetite or overeating?”	−0.017	0.059
Question 6: “Feeling bad about yourself—or that you are a failure or have let yourself or your family down?”	0.492	0.543
Question 7: “Trouble concentrating on things, such as reading the newspaper or watching television?”	−0.214	−0.213
Question 8: “Moving or speaking so slowly that other people could have noticed? Or the opposite—being so fidgety or restless that you have been moving around a lot more than usual?”	0.064	0.093
Question 9: “Thoughts that you would be better off dead, or of hurting yourself in some way?”	0.479	0.447
Question 1+Question 2+Question 6+Question 9	0.655	0.580
Question 2+Question 6+Question 9	0.727	0.698

^a^PHQ-9: Patient Health Questionnaire-9.

**Table 11:** Mean values of each cohort and *P* values from 1-tailed *t* tests of the top 5 important features in the random forest model for Patient Health Questionnaire-9 questions 2, 6, and 9.

Feature	Cohort, mean (SD)			*P* value
	None	Severe		
App 3 opens: number of times a user opened apps that fall into app category 3—active messaging and communication apps—within a 24-hour period (counts)	110.49 (73.47)	74.45 (77.25)	<.001	
App 3 upper: number of times a user opened apps that fall into app category 3—active messaging and communication apps—and had session times greater than the average session time of that app category within a 24-hour period (counts)	6.47 (5.77)	3.25 (3.98)	<.001	
App 2 opens: number of times a user opened apps that fall into app category 2—passive information and consumption apps—within a 24-hour period (counts)	2.13 (5.24)	0.46 (1.2)	<.001	
App 6: average time a user spent on apps that fall into app category 6—general utilities apps—within a 24-hour period (minutes)	0.40 (2.11)	0.57 (0.48)	<.001	
App 2: average time a user spent on apps that fall into app category 2—passive information and consumption apps—within a 24-hour period (minutes)	0.29 (0.70)	0.18 (0.20)	.008	

## Discussion

### Principal Findings

The study objective was to demonstrate a novel machine learning mental behavioral profiling metric termed MHSS, derived from analyzing passively monitored and nonintrusive smartphone use data, to identify and track depressive behavior. This objective was met as the MHSS models reached an overall accuracy of 87%. In this study, an average of 10 days of smartphone data were used in addition to PHQ-9 results from 399 participants to demonstrate the ability to detect digital behavioral markers quantified from the participants’ smartphones to detect depression severity. We further focused on using these digital behavioral markers to develop predictive models to classify *none* (not depressed) and *severe* (depressed) symptom severity scores.

A mental behavioral profiling metric termed MHSS, developed from digital markers extracted from the participants’ smartphone data, was able to predict the participants’ depression state (ie, none or severe) with high predictive performance using machine learning models.

Demographic analysis found a higher number of women in the *severe* group compared with the *none* group, which is in line with previous literature on the prevalence of depression in South Korea [46]. Studies conducted in South Korea have shown that, in a sample, women were more depressed than men across all age groups [47]. Both regression (random forest regression and multivariate adaptive regression splines) and classification (random forest classification, extreme gradient boosting, and support vector machines) machine learning models were tested to evaluate the highest predictive accuracy between none and severe depression. The PHQ-9 binary nonsensor model (none vs severe) achieved the highest accuracy using a random forest classification algorithm with the following metrics: precision of 85% to 89%, recall of 85% to 89%, F_1_ of 87%, and overall accuracy of 87%. The PHQ-9 3-class (none vs mild vs severe) model achieved the following metrics: precision of 74% to 86%, recall of 76% to 83%, F_1_ of 75% to 84%, and overall accuracy of 78%. The effect size of the nonsensor features was moderate, and the effect size of the sensor features was low. The PHQ-9 gyroscope sensor model achieved the following metrics: precision of 74% to 78%, recall of 67% to 83%, F_1_ of 72% to 78%, and overall accuracy of 76%. Although the results of this study have similar accuracies to previous studies [24,48], these models indicate that invasive features such as GPS tracking and audio information are not necessarily required to detect behaviors in individuals with depression.

The feature importance list was extracted based on the Gini Impurity measurement. In the PHQ-9 binary nonsensor model, results found that *mean session time* was the most important feature in predicting severe depression using nonintrusive passive sensors. Mean session time was higher in participants with *severe depression* compared with *none*. This result is in line with previous findings that people with higher phone use have a positive correlation with self-reported depression [49]. Another study found that high mobile phone use was associated with symptoms of depression in men and women at 1-year follow-up compared with people with low phone use [50]. Going down the feature importance plot, it was observed that app category 1 (social interaction apps) had higher use in participants with severe depression compared with participants with no depression. This aligns with studies that have shown that social media use is higher in people with depression [51,52] and that limited use can lead to a decline in self-reported feelings of depression [53]. In the PHQ-9 questions model, it was interesting to find that mean session time on active messaging and communication apps was lower in participants with *severe* depression (ie, their use of this app category was low compared with the *none* group). This finding is in line with previous findings that web-based communication is reported to be low in people with depression [54].

A per-item correlation was performed, and a significant positive Pearson correlation was found between PHQ-9 questions 2, 6, and 9 within the *severe* category users and the mental health behavioral profiling metric, that is, the MHSS (*r*=0.73). Users who had higher scores in the 3 questions also had higher MHSSs (>50%). Previous research has shown that items 2, 6, and 9 comprise the affective-cognitive component of the PHQ-9 scale [55]. The highest-correlated PHQ-9 items—2, 6, and 9—were the questions that indicated affective symptomatology; therefore, a separate PHQ-9 questions model was created, achieving a precision of 76% to 80%, recall of 75% to 81%, F_1_ of 78% to 89%, and overall accuracy of 78%. This 2D PHQ-9 questionnaire (the other being the somatic component) approach has been used in previous studies and shown to have sound psychometric reliability and validity [55-57]. Thus, the results of this study add to the literature as, to the best of our knowledge, no previous studies have explored the 2-factorial approach of the questionnaire and used it to create digital behavioral markers.

We chose South Korea as the study site as there are low levels of reported depression despite the high number of cases of suicide. The study found that, of the 469 individuals who reported having *no diagnosis* as their current status in their demographics questionnaire, 307 (65.5%) scored as moderate to severe depression (PHQ-9 score ≥10). This result fits previous literature that states that the population in South Korea is often less likely to seek treatment and diagnosis for depression because of low awareness and stigma [58]. Our results also complement our intention to study a South Korean sample as a previous study on the prevalence rates of depression in South Korea found that, despite the high suicide rate in the country [59], the prevalence of depression has been reported to be much lower compared with other countries [60]. This can be mainly due to 2 factors: the access rate to services for depression has been reported to be low and the mental health treatment gap for major depression is 56.3% [61]. It is also interesting that studies have shown that the prevalence of depression rates is lower in Asian countries, such as South Korea, when compared with Western countries [62,63] owing to the stigma surrounding psychiatric illnesses [64]. This result demonstrates the feasibility of a daily mental health profiling metric using smartphone-based passive data to monitor symptoms, administer tests at home, and schedule interventions, which will help overcome the limitations hindering traditional methods of assessment such as stigma [64] and hesitation toward accessing mental health services because of low education levels [58].

This study also evaluated the use of another passive sensor (ie, gyroscope) to improve the accuracy of our models. This study found that, when a gyroscope sensor was added as a feature, the Pearson correlation among questions 2, 6, and 9 decreased from 0.73 to 0.46. Mean activity (*P*<.001) and average gap activity (*P*<.001) features from the gyroscope sensors showed statistically significant differences between *none* and *severe* individuals. Therefore, although gyroscope sensor data show some distinction between the 2 cohorts when including them as an additional feature, the gyroscope as a sensor alone does not add predictive power.

Previous researchers have established a relationship between depression and digital phenotyping using identifiable passive information such as GPS and HealthKit information [8,48] and have a high respondent burden, such as daily mood surveys and multiple assessments [24,48]. Our study adds an approach in which we show that high-accuracy models to detect depression can be achieved using nonintrusive data such as average time on the phone per day, frequency of events per day, and app category use per day, with only 1 baseline assessment and no further respondent burden. The behavioral profiling metric, called the MHSS, is easy to understand by the user and, therefore, is easily incorporated into various clinical and therapeutic scenarios. The findings about various app category uses provide a dive into behavior patterns of depressed and not depressed groups, which can be useful for risk profiling. This study also further complements the idea shared by Onnela [65] in his research that private passive data collected from smartphones present a big challenge and should be anonymized. The app used in this study collects only nonintrusive, passive data, and the data are encrypted from the time they are collected and then re-encrypted when they are stored in the servers, thereby guaranteeing an accurate and safe MHSS. To further address any concerns about security, the app provides the ability to obtain daily MHSSs as a completely anonymous user, ensuring zero traceability.

In the everyday clinical scenario, the MHSS can help with remote monitoring of symptoms as well as treatment or intervention efficacy. It is a simple, affordable, and accessible form of technology that is easily scalable. This proves especially useful in low- and middle-income countries, where there are multiple barriers to mental health care access. In our study, we found that the MHSS can detect individual patterns of behavior as well as population-based trends. However, further research is required to establish its use on an epidemiological level.

### Strengths

The strength of the study can be found in using nonintrusive, passive behavioral data to generate digital phenotypes for depression and, in the future, for more mental health disorders. In addition, web-based recruitment was used, which eased the onboarding process and allowed the users to participate in the study at their own comfort. This study design is easy to replicate for other digital phenotyping indications where it is possible to administer web-based self-report questionnaires and generate results.

### Limitations and Future Work

The first limitation of the study was that the PHQ-9 was administered only once; thus, the depression symptom status was only collected at baseline. Future studies should aim to assess the symptoms at 2 time points and observe the changes in questionnaire scores alongside the changes in digital behavior. Another limitation was that our data were heavily inclined toward the 18 to 25-year age group, with 84.9% (474/558) of the participants belonging to it. Our study did not have *clinical diagnosis* *of depression* as an inclusion criterion, only a self-reported clinical diagnosis and the self-reported PHQ-9 scale. Using a more diverse age group in a more proportionate number could provide a better overview of how digital behavior symptom severity could change with age as a factor. Although the PHQ-9 as a patient-reported outcome measure is the gold standard method for diagnosing depression and is used worldwide to screen for depression, the results of this study will be further consolidated when tested in a clinically diagnosed population. This study was available only for Android users; therefore, further studies should look at incorporating the iOS operating system. Furthermore, future studies can include other locations and questionnaires.

### Conclusions

Nonidentifiable passive smartphone data prove to be a suitable tool to assist with the remote screening and monitoring of depression. The strong privacy metrics and low respondent burden pave the way for further exploration in not only screening and even triaging patients but also measuring therapeutic outcomes through the MHSS as a metric. Finally, the aggregated measurement of a group as a health metric could further support larger epidemiological studies.

## Appendix

Multimedia Appendix 1 Behavidence app categories.

Multimedia Appendix 2 Full list of 37 digital features extracted from the passive data collected in the study with their description.

Multimedia Appendix 3 List of features and their significance. Of 37 features, 8 significant sensor and nonsensors are displayed in the text. This is the list of the remaining 29 features and their significance.

## Abbreviations

DSM-5: Diagnostic and Statistical Manual of Mental Disorders, Fifth Edition
MHSS: Mental Health Similarity Score
PHQ-9: Patient Health Questionnaire-9

## References

[1] What is depression. American Psychiatric Association.

[2] Kanter JW, Busch AM, Weeks CE, Landes SJ (2017). The nature of clinical depression: symptoms, syndromes, and behavior analysis. Behav Analyst.

[3] Vos T, Lim SS, Abbafati C, Abbas KM, Abbasi M, Abbasifard M, Abbasi-Kangevari M, Abbastabar H, Abd-Allah F, Abdelalim A, Abdollahi M, Abdollahpour I, Abolhassani H, Aboyans V, Abrams E, Abreu L, Abrigo M, Abu-Raddad L, Abushouk A, Acebedo A, Ackerman I, Adabi M, Adamu A, Adebayo O, Adekanmbi V, Adelson J, Adetokunboh O, Adham D, Afshari M, Afshin A, Agardh E, Agarwal G, Agesa K, Aghaali M, Aghamir S, Agrawal A, Ahmad T, Ahmadi A, Ahmadi M, Ahmadieh H, Ahmadpour E, Akalu T, Akinyemi R, Akinyemiju T, Akombi B, Al-Aly Z, Alam K, Alam N, Alam S, Alam T, Alanzi T, Albertson S, Alcalde-Rabanal J, Alema N, Ali M, Ali S, Alicandro G, Alijanzadeh M, Alinia C, Alipour V, Aljunid S, Alla F, Allebeck P, Almasi-Hashiani A, Alonso J, Al-Raddadi R, Altirkawi K, Alvis-Guzman N, Alvis-Zakzuk N, Amini S, Amini-Rarani M, Aminorroaya A, Amiri F, Amit A, Amugsi D, Amul G, Anderlini D, Andrei C, Andrei T, Anjomshoa M, Ansari F, Ansari I, Ansari-Moghaddam A, Antonio C, Antony C, Antriyandarti E, Anvari D, Anwer R, Arabloo J, Arab-Zozani M, Aravkin A, Ariani F, Ärnlöv J, Aryal K, Arzani A, Asadi-Aliabadi M, Asadi-Pooya A, Asghari B, Ashbaugh C, Atnafu D, Atre S, Ausloos F, Ausloos M, Ayala Quintanilla B, Ayano G, Ayanore M, Aynalem Y, Azari S, Azarian G, Azene Z, Babaee E, Badawi A, Bagherzadeh M, Bakhshaei M, Bakhtiari A, Balakrishnan S, Balalla S, Balassyano S, Banach M, Banik P, Bannick M, Bante A, Baraki A, Barboza M, Barker-Collo S, Barthelemy C, Barua L, Barzegar A, Basu S, Baune B, Bayati M, Bazmandegan G, Bedi N, Beghi E, Béjot Y, Bello A, Bender R, Bennett D, Bennitt F, Bensenor I, Benziger C, Berhe K, Bernabe E, Bertolacci G, Bhageerathy R, Bhala N, Bhandari D, Bhardwaj P, Bhattacharyya K, Bhutta Z, Bibi S, Biehl M, Bikbov B, Bin Sayeed M, Biondi A, Birihane B, Bisanzio D, Bisignano C, Biswas R, Bohlouli S, Bohluli M, Bolla S, Boloor A, Boon-Dooley A, Borges G, Borzì A, Bourne R, Brady O, Brauer M, Brayne C, Breitborde N, Brenner H, Briant P, Briggs A, Briko N, Britton G, Bryazka D, Buchbinder R, Bumgarner B, Busse R, Butt Z, Caetano dos Santos F, Cámera L, Campos-Nonato I, Car J, Cárdenas R, Carreras G, Carrero J, Carvalho F, Castaldelli-Maia J, Castañeda-Orjuela C, Castelpietra G, Castle C, Castro F, Catalá-López F, Causey K, Cederroth C, Cercy K, Cerin E, Chandan J, Chang A, Charlson F, Chattu V, Chaturvedi S, Chimed-Ochir O, Chin K, Cho D, Christensen H, Chu D, Chung M, Cicuttini F, Ciobanu L, Cirillo M, Collins E, Compton K, Conti S, Cortesi P, Costa V, Cousin E, Cowden R, Cowie B, Cromwell E, Cross D, Crowe C, Cruz J, Cunningham M, Dahlawi S, Damiani G, Dandona L, Dandona R, Darwesh A, Daryani A, Das J, Das Gupta R, das Neves J, Dávila-Cervantes C, Davletov K, De Leo D, Dean F, DeCleene N, Deen A, Degenhardt L, Dellavalle R, Demeke F, Demsie D, Denova-Gutiérrez E, Dereje N, Dervenis N, Desai R, Desalew A, Dessie G, Dharmaratne S, Dhungana G, Dianatinasab M, Diaz D, Dibaji Forooshani Z, Dingels Z, Dirac M, Djalalinia S, Do H, Dokova K, Dorostkar F, Doshi C, Doshmangir L, Douiri A, Doxey M, Driscoll T, Dunachie S, Duncan B, Duraes A, Eagan A, Ebrahimi Kalan M, Edvardsson D, Ehrlich J, El Nahas N, El Sayed I, El Tantawi M, Elbarazi I, Elgendy I, Elhabashy H, El-Jaafary S, Elyazar I, Emamian M, Emmons-Bell S, Erskine H, Eshrati B, Eskandarieh S, Esmaeilnejad S, Esmaeilzadeh F, Esteghamati A, Estep K, Etemadi A, Etisso A, Farahmand M, Faraj A, Fareed M, Faridnia R, Farinha C, Farioli A, Faro A, Faruque M, Farzadfar F, Fattahi N, Fazlzadeh M, Feigin V, Feldman R, Fereshtehnejad S, Fernandes E, Ferrari A, Ferreira M, Filip I, Fischer F, Fisher J, Fitzgerald R, Flohr C, Flor L, Foigt N, Folayan M, Force L, Fornari C, Foroutan M, Fox J, Freitas M, Fu W, Fukumoto T, Furtado J, Gad M, Gakidou E, Galles N, Gallus S, Gamkrelidze A, Garcia-Basteiro A, Gardner W, Geberemariyam B, Gebrehiwot A, Gebremedhin K, Gebreslassie A, Gershberg Hayoon A, Gething P, Ghadimi M, Ghadiri K, Ghafourifard M, Ghajar A, Ghamari F, Ghashghaee A, Ghiasvand H, Ghith N, Gholamian A, Gilani S, Gill P, Gitimoghaddam M, Giussani G, Goli S, Gomez R, Gopalani S, Gorini G, Gorman T, Gottlich H, Goudarzi H, Goulart A, Goulart B, Grada A, Grivna M, Grosso G, Gubari M, Gugnani H, Guimaraes A, Guimarães R, Guled R, Guo G, Guo Y, Gupta R, Haagsma J, Haddock B, Hafezi-Nejad N, Hafiz A, Hagins H, Haile L, Hall B, Halvaei I, Hamadeh R, Hamagharib Abdullah K, Hamilton E, Han C, Han H, Hankey G, Haro J, Harvey J, Hasaballah A, Hasanzadeh A, Hashemian M, Hassanipour S, Hassankhani H, Havmoeller R, Hay R, Hay S, Hayat K, Heidari B, Heidari G, Heidari-Soureshjani R, Hendrie D, Henrikson H, Henry N, Herteliu C, Heydarpour F, Hird T, Hoek H, Hole M, Holla R, Hoogar P, Hosgood H, Hosseinzadeh M, Hostiuc M, Hostiuc S, Househ M, Hoy D, Hsairi M, Hsieh V, Hu G, Huda T, Hugo F, Huynh C, Hwang B, Iannucci V, Ibitoye S, Ikuta K, Ilesanmi O, Ilic I, Ilic M, Inbaraj L, Ippolito H, Irvani S, Islam M, Islam M, Islam S, Islami F, Iso H, Ivers R, Iwu C, Iyamu I, Jaafari J, Jacobsen K, Jadidi-Niaragh F, Jafari H, Jafarinia M, Jahagirdar D, Jahani M, Jahanmehr N, Jakovljevic M, Jalali A, Jalilian F, James S, Janjani H, Janodia M, Jayatilleke A, Jeemon P, Jenabi E, Jha R, Jha V, Ji J, Jia P, John O, John-Akinola Y, Johnson C, Johnson S, Jonas J, Joo T, Joshi A, Jozwiak J, Jürisson M, Kabir A, Kabir Z, Kalani H, Kalani R, Kalankesh L, Kalhor R, Kamiab Z, Kanchan T, Karami Matin B, Karch A, Karim M, Karimi S, Kassa G, Kassebaum N, Katikireddi S, Kawakami N, Kayode G, Keddie S, Keller C, Kereselidze M, Khafaie M, Khalid N, Khan M

[4] Greenberg PE, Fournier A, Sisitsky T, Simes M, Berman R, Koenigsberg SH, Kessler RC (2021). The economic burden of adults with major depressive disorder in the United States (2010 and 2018). Pharmacoeconomics.

[5] Nochaiwong S, Ruengorn C, Thavorn K, Hutton B, Awiphan R, Phosuya C, Ruanta Y, Wongpakaran N, Wongpakaran T (2021). Global prevalence of mental health issues among the general population during the coronavirus disease-2019 pandemic: a systematic review and meta-analysis. Sci Rep.

[6] New Global Burden of Disease analyses show depression and anxiety among the top causes of health loss worldwide, and a significant increase due to the COVID-19 pandemic. IHME.

[7] Marroquín B, Vine V, Morgan R (2020). Mental health during the COVID-19 pandemic: effects of stay-at-home policies, social distancing behavior, and social resources. Psychiatry Res.

[8] Moshe I, Terhorst Y, Opoku Asare K, Sander LB, Ferreira D, Baumeister H, Mohr DC, Pulkki-Råback L (2021). Predicting symptoms of depression and anxiety using smartphone and wearable data. Front Psychiatry.

[9] (2013). The Diagnostic and Statistical Manual of Mental Disorders, Fifth Edition.

[10] ICD-11. World Health Organization.

[11] Newson J, Pastukh V, Thiagarajan T (2021). Poor separation of clinical symptom profiles by DSM-5 disorder criteria. Front Psychiatry.

[12] Montgomery S (2016). Are the ICD-10 or DSM-5 diagnostic systems able to define those who will benefit from treatment for depression?. CNS Spectr.

[13] Zimmerman M, Ellison W, Young D, Chelminski I, Dalrymple K (2015). How many different ways do patients meet the diagnostic criteria for major depressive disorder?. Compr Psychiatry.

[14] Fried EI (2017). The 52 symptoms of major depression: lack of content overlap among seven common depression scales. J Affect Disord.

[15] Ghosh CC, McVicar D, Davidson G, Shannon C (2021). Measuring diagnostic heterogeneity using text-mining of the lived experiences of patients. BMC Psychiatry.

[16] Ayano G, Demelash S, Yohannes Z, Haile K, Tulu M, Assefa D, Tesfaye A, Haile K, Solomon M, Chaka A, Tsegay L (2021). Misdiagnosis, detection rate, and associated factors of severe psychiatric disorders in specialized psychiatry centers in Ethiopia. Ann Gen Psychiatry.

[17] Hacimusalar Y, Eşel E (2018). Suggested biomarkers for major depressive disorder. Noro Psikiyatr Ars.

[18] Egede LE (2007). Failure to recognize depression in primary care: issues and challenges. J Gen Intern Med.

[19] Bukh JD, Bock C, Vinberg M, Kessing LV (2013). The effect of prolonged duration of untreated depression on antidepressant treatment outcome. J Affect Disord.

[20] Pedrelli P, Fedor S, Ghandeharioun A, Howe E, Ionescu DF, Bhathena D, Fisher LB, Cusin C, Nyer M, Yeung A, Sangermano L, Mischoulon D, Alpert JE, Picard RW (2020). Monitoring changes in depression severity using wearable and mobile sensors. Front Psychiatry.

[21] Depression assessment instruments. American Psychological Association.

[22] Kroenke K, Spitzer RL, Williams JB (2001). The PHQ-9: validity of a brief depression severity measure. J Gen Intern Med.

[23] Middelweerd A, Mollee JS, van der Wal CN, Brug J, Te Velde SJ (2014). Apps to promote physical activity among adults: a review and content analysis. Int J Behav Nutr Phys Act.

[24] Wahle F, Kowatsch T, Fleisch E, Rufer M, Weidt S (2016). Mobile sensing and support for people with depression: a pilot trial in the wild. JMIR Mhealth Uhealth.

[25] Torous J, Kiang MV, Lorme J, Onnela J-P (2016). New tools for new research in psychiatry: a scalable and customizable platform to empower data driven smartphone research. JMIR Ment Health.

[26] Torous J, Chan SR, Yee-Marie Tan S, Behrens J, Mathew I, Conrad EJ, Hinton L, Yellowlees P, Keshavan M (2014). Patient smartphone ownership and interest in mobile apps to monitor symptoms of mental health conditions: a survey in four geographically distinct psychiatric clinics. JMIR Ment Health.

[27] East ML, Havard BC (2015). Mental health mobile apps: from infusion to diffusion in the mental health social system. JMIR Ment Health.

[28] Torous J, Staples P, Shanahan M, Lin C, Peck P, Keshavan M, Onnela J (2015). Utilizing a personal smartphone custom app to assess the patient health questionnaire-9 (PHQ-9) depressive symptoms in patients with major depressive disorder. JMIR Ment Health.

[29] Saeb S, Zhang M, Karr CJ, Schueller SM, Corden ME, Kording KP, Mohr DC (2015). Mobile phone sensor correlates of depressive symptom severity in daily-life behavior: an exploratory study. J Med Internet Res.

[30] Marsch LA (2021). Digital health data-driven approaches to understand human behavior. Neuropsychopharmacology.

[31] Wang R, Chen F, Chen Z, Li T, Harari G, Tignor S, Zhou X, Ben-Zeev D, Campbell AT (2014). StudentLife: assessing mental health, academic performance and behavioral trends of college students using smartphones. Proceedings of the 2014 ACM International Joint Conference on Pervasive and Ubiquitous Computing.

[32] Saeb S, Zhang M, Kwasny M, Karr C, Kording K, Mohr D (2015). The relationship between clinical, momentary, and sensor-based assessment of depression. Proceedings of the 9th International Conference on Pervasive Computing Technologies for Healthcare.

[33] Opoku Asare K, Terhorst Y, Vega J, Peltonen E, Lagerspetz E, Ferreira D (2021). Predicting depression from smartphone behavioral markers using machine learning methods, hyperparameter optimization, and feature importance analysis: exploratory study. JMIR Mhealth Uhealth.

[34] Razavi R, Gharipour A, Gharipour M (2020). Depression screening using mobile phone usage metadata: a machine learning approach. J Am Med Inform Assoc.

[35] Goltermann J, Emden D, Leehr EJ, Dohm K, Redlich R, Dannlowski U, Hahn T, Opel N (2021). Smartphone-based self-reports of depressive symptoms using the remote monitoring application in psychiatry (ReMAP): interformat validation study. JMIR Ment Health.

[36] Lee S, Park J, Lee S, Oh I, Choi J, Oh C (2018). Changing trends in suicide rates in South Korea from 1993 to 2016: a descriptive study. BMJ Open.

[37] (2011). Epidemiology of depressive disorders. Textbook of Psychiatric Epidemiology, Third Edition.

[38] Melcher J, Hays R, Torous J (2020). Digital phenotyping for mental health of college students: a clinical review. Evid Based Ment Health.

[39] Gelinas L, Pierce R, Winkler S, Cohen IG, Lynch HF, Bierer BE (2017). Using social media as a research recruitment tool: ethical issues and recommendations. Am J Bioeth.

[40] Spitzer RL, Kroenke K, Williams JB (1999). Validation and utility of a self-report version of PRIME-MD: the PHQ primary care study. Primary care evaluation of mental disorders. Patient health questionnaire. JAMA.

[41] Sun Y, Kong Z, Song Y, Liu J, Wang X (2022). The validity and reliability of the PHQ-9 on screening of depression in neurology: a cross sectional study. BMC Psychiatry.

[42] Levis B, Benedetti A, Thombs BD, DEPRESsion Screening Data (DEPRESSD) Collaboration (2019). Accuracy of Patient Health Questionnaire-9 (PHQ-9) for screening to detect major depression: individual participant data meta-analysis. BMJ.

[43] Shin C, Ko Y, An H, Yoon H, Han C (2020). Normative data and psychometric properties of the patient health questionnaire-9 in a nationally representative Korean population. BMC Psychiatry.

[44] How are you today? Get your daily mental health score. Behavidence.

[45] Schäfer T, Schwarz M (2019). The meaningfulness of effect sizes in psychological research: differences between sub-disciplines and the impact of potential biases. Front Psychol.

[46] Kim GE, Jo M, Shin Y (2020). Increased prevalence of depression in South Korea from 2002 to 2013. Sci Rep.

[47] Shin C, Kim Y, Park S, Yoon S, Ko Y, Kim Y, Kim S, Jeon SW, Han C (2017). Prevalence and associated factors of depression in general population of Korea: results from the Korea national health and nutrition examination survey, 2014. J Korean Med Sci.

[48] Sarda A, Munuswamy S, Sarda S, Subramanian V (2019). Using passive smartphone sensing for improved risk stratification of patients with depression and diabetes: cross-sectional observational study. JMIR Mhealth Uhealth.

[49] Alhassan AA, Alqadhib EM, Taha NW, Alahmari RA, Salam M, Almutairi AF (2018). The relationship between addiction to smartphone usage and depression among adults: a cross sectional study. BMC Psychiatry.

[50] Thomée S, Härenstam A, Hagberg M (2011). Mobile phone use and stress, sleep disturbances, and symptoms of depression among young adults-a prospective cohort study. BMC Public Health.

[51] Donnelly E (2017). Depression among users of Social Networking Sites (SNSs): the role of SNS addiction and increased usage. J Addict Prev Med.

[52] Perlis RH, Green J, Simonson M, Ognyanova K, Santillana M, Lin J, Quintana A, Chwe H, Druckman J, Lazer D, Baum MA, Della Volpe J (2021). Association between social media use and self-reported symptoms of depression in US adults. JAMA Netw Open.

[53] Hunt MG, Marx R, Lipson C, Young J (2018). No more FOMO: limiting social media decreases loneliness and depression. J Soc Clin Psychol.

[54] Nakagomi A, Shiba K, Kondo K, Kawachi I (2022). Can online communication prevent depression among older people? A longitudinal analysis. J Appl Gerontol.

[55] Hinz A, Mehnert A, Kocalevent R, Brähler E, Forkmann T, Singer S, Schulte T (2016). Assessment of depression severity with the PHQ-9 in cancer patients and in the general population. BMC Psychiatry.

[56] Chilcot J, Rayner L, Lee W, Price A, Goodwin L, Monroe B, Sykes N, Hansford P, Hotopf M (2013). The factor structure of the PHQ-9 in palliative care. J Psychosom Res.

[57] Elhai JD, Contractor AA, Tamburrino M, Fine TH, Prescott MR, Shirley E, Chan PK, Slembarski R, Liberzon I, Galea S, Calabrese JR (2012). The factor structure of major depression symptoms: a test of four competing models using the patient health questionnaire-9. Psychiatry Res.

[58] Jang J, Lee SA, Kim W, Choi Y, Park E (2018). Factors associated with mental health consultation in South Korea. BMC Psychiatry.

[59] (2015). Health at a Glance 2015 : OECD Indicators.

[60] Rotenstein LS, Ramos MA, Torre M, Segal JB, Peluso MJ, Guille C, Sen S, Mata DA (2016). Prevalence of depression, depressive symptoms, and suicidal ideation among medical students: a systematic review and meta-analysis. JAMA.

[61] Kohn R, Saxena S, Levav I, Saraceno B (2004). The treatment gap in mental health care. Bull World Health Organ.

[62] Bromet E, Andrade LH, Hwang I, Sampson NA, Alonso J, de Girolamo G, de Graaf R, Demyttenaere K, Hu C, Iwata N, Karam AN, Kaur J, Kostyuchenko S, Lépine JP, Levinson D, Matschinger H, Mora ME, Browne MO, Posada-Villa J, Viana MC, Williams DR, Kessler RC (2011). Cross-national epidemiology of DSM-IV major depressive episode. BMC Med.

[63] Lim GY, Tam WW, Lu Y, Ho CS, Zhang MW, Ho RC (2018). Prevalence of depression in the community from 30 countries between 1994 and 2014. Sci Rep.

[64] Zhang Z, Sun K, Jatchavala C, Koh J, Chia Y, Bose J, Li Z, Tan W, Wang S, Chu W, Wang J, Tran B, Ho R (2019). Overview of stigma against psychiatric illnesses and advancements of anti-stigma activities in six Asian societies. Int J Environ Res Public Health.

[65] Onnela J (2021). Opportunities and challenges in the collection and analysis of digital phenotyping data. Neuropsychopharmacology.

