# Supplementary material for: A Machine Learning Approach for Detecting Digital Behavioral Patterns of Depression Using Nonintrusive Smartphone Data (Complementary Path to Patient Health Questionnaire-9 Assessment): Prospective Observational Study
Source: JMIR Form Res. 2022 May 16;6(5):e37736. doi: 10.2196/37736 (PMC9152726; doi:10.2196/37736)
Supplement: Multimedia Appendix 1 [file formative_v6i5e37736_app1.docx]

Appendix 1

**Behavidence App Categories:**

Category 0 - Non-official apps or unregulated apps

Category 1 - Social interaction applications

Category 2 - Passive information consumption apps

Category 3 - Active Messaging and Communications

Category 4 - Educational apps

Category 5 - Navigation utilities

Category 6 - General utilities

Category 7 - Recreational and Photo processing

Category 8 - Commerce

Category 9 - Health & Fitness

Category 10 - Games

Category 11 - Miscellaneous and additional Passive recreational apps
