# Supplementary material for: A Machine Learning Approach for Detecting Digital Behavioral Patterns of Depression Using Nonintrusive Smartphone Data (Complementary Path to Patient Health Questionnaire-9 Assessment): Prospective Observational Study
Source: JMIR Form Res. 2022 May 16;6(5):e37736. doi: 10.2196/37736 (PMC9152726; doi:10.2196/37736)
Supplement: Multimedia Appendix 2 [file formative_v6i5e37736_app2.docx]

### Appendix 2. Full list of 37 digital features extracted from the passive data collected in the study with their description.

| **Feature** | **Definition** |
| --- | --- |
| Mean session | Average session length a user interacts with their mobile device within a 24-hour period (minutes) |
| Total session | Sum of session lengths a user interacts with their mobile device within a 24-hour period (minutes) |
| Number of opens | Number of times a user opens an app within a 24-hour period |
| Sleep | Longest gap time a user is not interacting with their phone within a 24-hour period (minutes) |
| Average gap | Average length of time a user is not interacting with their phone within a 24-hour period (minutes) |
| App 0 | Average time a user spent on apps that fall into app category 0 within a 24-hour period (minutes) |
| App 1 | Average time a user spent on apps that fall into app category 1 within a 24-hour period (minutes) |
| App 2 | Average time a user spent on apps that fall into app category 2 within a 24-hour period (minutes) |
| App 3 | Average time a user spent on apps that fall into app category 3 within a 24-hour period (minutes) |
| App 5 | Average time a user spent on apps that fall into app category within a 24-hour period (minutes) |
| App 6 | Average time a user spent on apps that fall into app category within a 24-hour period (minutes) |
| App 7 | Average time a user spent on apps that fall into app category 7 within a 24-hour period (minutes) |
| App 8 | Average time a user spent on apps that fall into app category 8 within a 24-hour period (minutes) |
| App 9 | Average time a user spent on apps that fall into app category within a 24-hour period (minutes) |
| App 10 | Average time a user spent on apps that fall into app category within a 24-hour period (minutes) |
| App 11 | Average time a user spent on apps that fall into app category 11 within a 24-hour period (minutes) |
| App 1- Number of opens | Number of times a user opened apps that fall into app category 1 within a 24-hour period |
| App 2- Number of opens | Number of times a user opened apps that fall into app category 2 within a 24-hour period |
| App 3- Number of opens | Number of times a user opened apps that fall into app category 3 within a 24-hour period |
| App 5- Number of opens | Number of times a user opened apps that fall into app category 5 within a 24-hour period |
| App 6- Number of opens | Number of times a user opened apps that fall into app category 6 within a 24-hour period |
| App 7- Number of opens | Number of times a user opened apps that fall into app category 7 within a 24-hour period |
| App 8- Number of opens | Number of times a user opened apps that fall into app category 8 within a 24-hour period |
| App 9- Number of opens | Number of times a user opened apps that fall into app category 9 within a 24-hour period |
| App 10- Number of opens | Number of times a user opened apps that fall into app category 10 within a 24-hour period |
| App 1- Upper Limit | Number of times a user opened apps that fall into app category 1 and had session times greater than the average session time of that app category within a 24-hour period |
| App 2- Upper Limit | Number of times a user opened apps that fall into app category 2 and had session times greater than the average session time of that app category within a 24-hour period |
| App 3- Upper Limit | Number of times a user opened apps that fall into app category 3 and had session times greater than the average session time of that app category within a 24-hour period |
| App 5- Upper Limit | Number of times a user opened apps that fall into app category 5 and had session times greater than the average session time of that app category within a 24-hour period |
| App 6- Upper Limit | Number of times a user opened apps that fall into app category 6 and had session times greater than the average session time of that app category within a 24-hour period |
| App 7- Upper Limit | Number of times a user opened apps that fall into app category 7 and had session times greater than the average session time of that app category within a 24-hour period |
| App 8- Upper Limit | Number of times a user opened apps that fall into app category 8 and had session times greater than the average session time of that app category within a 24-hour period |
| App 9- Upper Limit | Number of times a user opened apps that fall into app category 9 and had session times greater than the average session time of that app category within a 24-hour period |
| App 10- Upper Limit | Number of times a user opened apps that fall into app category 10 and had session times greater than the average session time of that app category within a 24-hour period |
| Average Activity | Average time the gyroscope sensed user movement within a 24-hour period (minutes) |
| Average Gap Activity | Average time the gyroscope sensed no user movement within a 24-hour period (minutes) |
| Total Activity | Total time the gyroscope sensed user movement within a 24-hour period (minutes) |
