# Supplementary material for: A Machine Learning Approach for Detecting Digital Behavioral Patterns of Depression Using Nonintrusive Smartphone Data (Complementary Path to Patient Health Questionnaire-9 Assessment): Prospective Observational Study
Source: JMIR Form Res. 2022 May 16;6(5):e37736. doi: 10.2196/37736 (PMC9152726; doi:10.2196/37736)
Supplement: Multimedia Appendix 3 [file formative_v6i5e37736_app3.docx]

**Appendix 3.** List of features and their significance. Of 37 features, 8 significant sensor and nonsensors are displayed in the text. This is the list of the remaining 29 features and their significance.

|  | Feature | Cohort    (Means) | | *P*-value | Cohen *d* |
| --- | --- | --- | --- | --- | --- |
| Non-Sensor |  |  |  |  |  |
|  |  | None | Severe |  |  |
|  | App 0 | 0.3  (0.6) | 0.8  (2.4) | *P*<0.001 | 0.2980 |
|  | App 1 | 1.4 (1.2) | 3.5 (4.3) | *P*<.001 | 0.7107 |
|  | App 2 | 0.3 (0.6) | 0.2 (1.1) | *P*=0.02 | 0.1248 |
|  | App 3 | 1.0 (0.6) | 1.3 (1.2) | *P*<.001 | 0.3395 |
|  | App 5 | 0.4(2.0) | 0.1  (0.8) | *P*=0.001 | 0.1554 |
|  | App 6 | 0.4 (0.5) | 0.6 (1.3) | *P*=0.002 | 0.1777 |
|  | App 7 | 0.5 (0.8) | 1.0 (3.5) | *P*<.001 | 0.2116 |
|  | App 8 | 0.4 (1.5) | 0.7 (1.8) | *P*<.001 | 0.1769 |
|  | App 9 | 0.4 (0.5) | 0.8 (2.8) | *P*= .002 | 0.1769 |
|  | App 10 | 0.8 (3.2) | 3.2 (9.0) | *P*<.001 | 0.3639 |
|  | App 11 | 1.6 (2.0) | 3.3 (5.2) | *P*<.001 | 0.4899 |
|  | App 1- Number of opens | 34.5 (34.7) | 29.7 (37.7) | *P*= .01 | 0.1338 |
|  | App 2- Number of opens | 2.1 (4.9) | 0.5 (2.4) | *P*<.001 | 0.4216 |
|  | App 3- Number of open | 110.4 (70.1) | 74.4 (71.0) | *P*<.001 | 0.5109 |
|  | App 5- Number of opens | 2.6 (6.4) | 0.4 (2.6) | *P*<.001 | 0.4464 |
|  | App 6- Number of opens | 13.5 (16.6) | 13.5 (27.4) | *P*= .99 | 0.0003 |
|  | App 7- Number of opens | 5.9 (9.5) | 4.3 (8.4) | *P*<.001 | 0.1697 |
|  | App 8- Number of opens | 1.9 (4.7) | 3.1(9.9) | *P=0.003* | 0.1678 |
|  | App 9- Number of opens | 6.5(7.3) | 5.4(17.8) | *P=0.15* | 0.0822 |
|  | App 10- Number of opens | 5.9(13.6) | 5.6(15.8) | *P=0.66* | 0.0233 |
|  | App 1- Upper Limit | 4.1 (4.7) | 3.8 (4.6) | *P=0.16* | 0.0734 |
|  | App 2- Upper Limit | 0.1 (0.4) | 0.1(0.6) | *P=0.55* | 0.0326 |
|  | App 3- Upper Limit | 6.5 (5.8) | 3.3 (4.8) | *P*<.001 | 0.5971 |
|  | App 5- Upper Limit | 0.1 (0.7) | 0.0 (0.4) | *P<0.001* | 0.1760 |
|  | App 6- Upper Limit | 0.3 (0.9) | 0.2 (0.7) | *P=0.02* | 0.1198 |
|  | App 7- Upper Limit | 0.1 (0.5) | 0.2 (0.8) | *P*= .05 | 0.1061 |
|  | App 8- Upper Limit | 0.2(0.7) | 0.3(1.3) | *P=0.02* | 0.1317 |
|  | App 9- Upper Limit | 0.2(0.6) | 0.2(0.7) | *P=0.51* | 0.0349 |
|  | App 10- Upper Limit | 0.6(1.6) | 1.2(3.5) | *P*<.001 | 0.2259 |
|  |  |  |  |  |  |
